# Supplementary material for: Quantum – coherent dynamics in photosynthetic charge separation revealed by wavelet analysis
Source: Sci Rep. 2017 Jun 6;7:2890. doi: 10.1038/s41598-017-02906-7 (PMC5460264; doi:10.1038/s41598-017-02906-7)
Supplement: Supplementary file 1 — Supplementary Information [file 41598_2017_2906_MOESM1_ESM.pdf]

Supplementary Information for

Quantum – coherent dynamics in photosynthetic charge separation revealed by wavelet analysis

Elisabet Romero[‡], Javier Prior[‡], Alex W. Chin, Sarah E. Morgan, Vladimir I. Novoderezhkin, Martin B. Plenio, and Rienk van Grondelle

[‡] These authors contributed equally to this work.

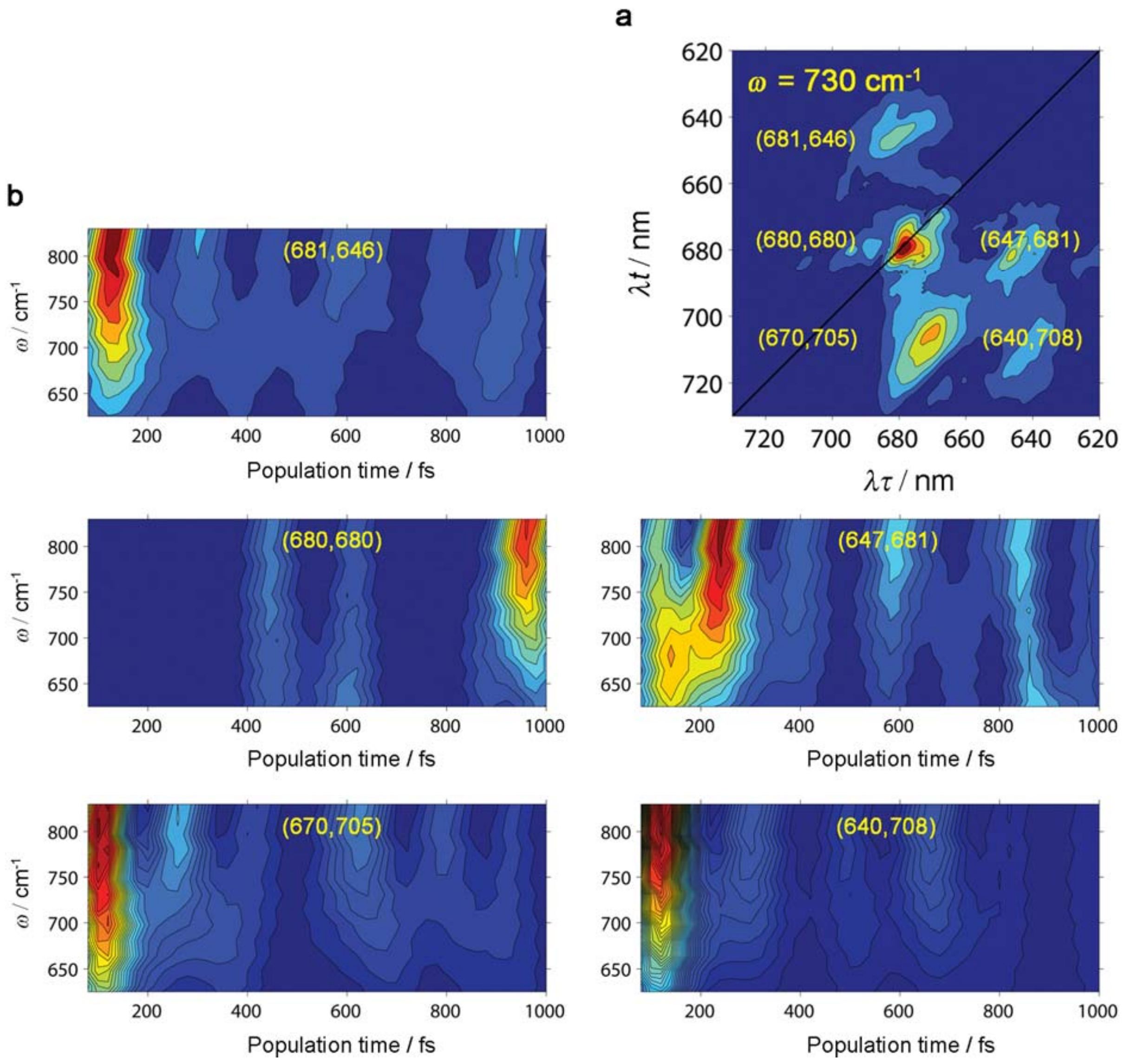

Figure S1. Vibrational coherence dynamics in the PSII RC at 80 K for the 730 cm^{-1} 2D frequency map. (**a**) The 2D frequency map at 730 cm^{-1} (this panel has been adapted from ref. 12 in the main text). (**b**) Experimental wavelet scalograms for the most intense 2D frequency map features.

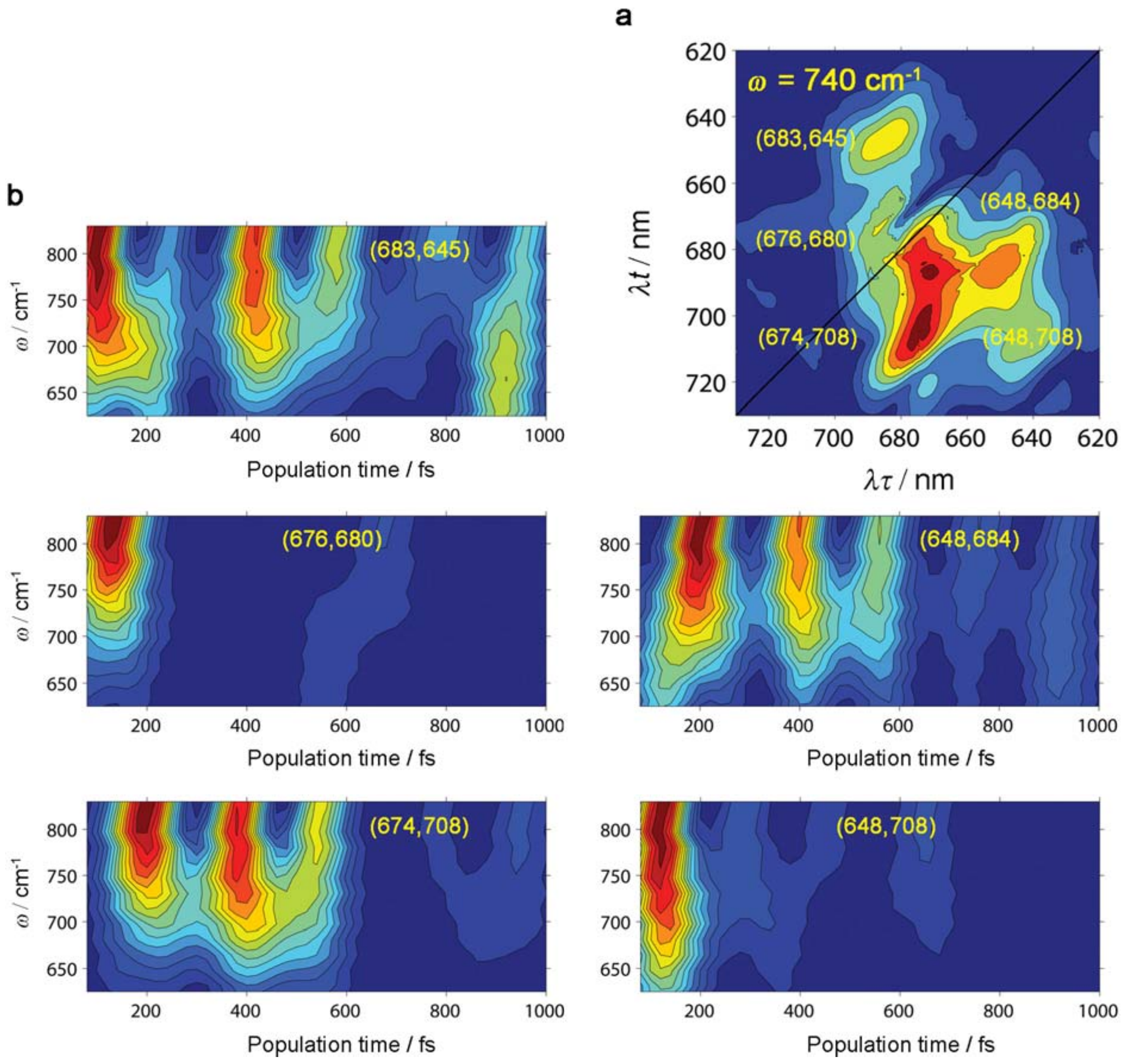

Figure S2. Vibrational coherence dynamics in the PSII RC at 277 K for the 740 cm^{-1} 2D frequency map. (**a**) The 2D frequency map at 730 cm^{-1} (this panel has been adapted from ref. 12 in the main text) (**b**) Experimental wavelet scalograms for the most intense 2D frequency map features.

The 730 cm^{-1} vibrational mode is far from all exciton splittings, however, the electronic (exciton and CTs) states have vibrational satellites separated from the zero-phonon level (ZPL) by values close to 730 cm^{-1} (which are affected by the exciton couplings) . As a result, immediately after excitation we observe several states oscillating in phase around 730 cm^{-1} which produce an intense peak at short times (within 300 fs). At longer times, these coherent oscillations decrease in amplitude due to environment-induced dephasing[1-2].

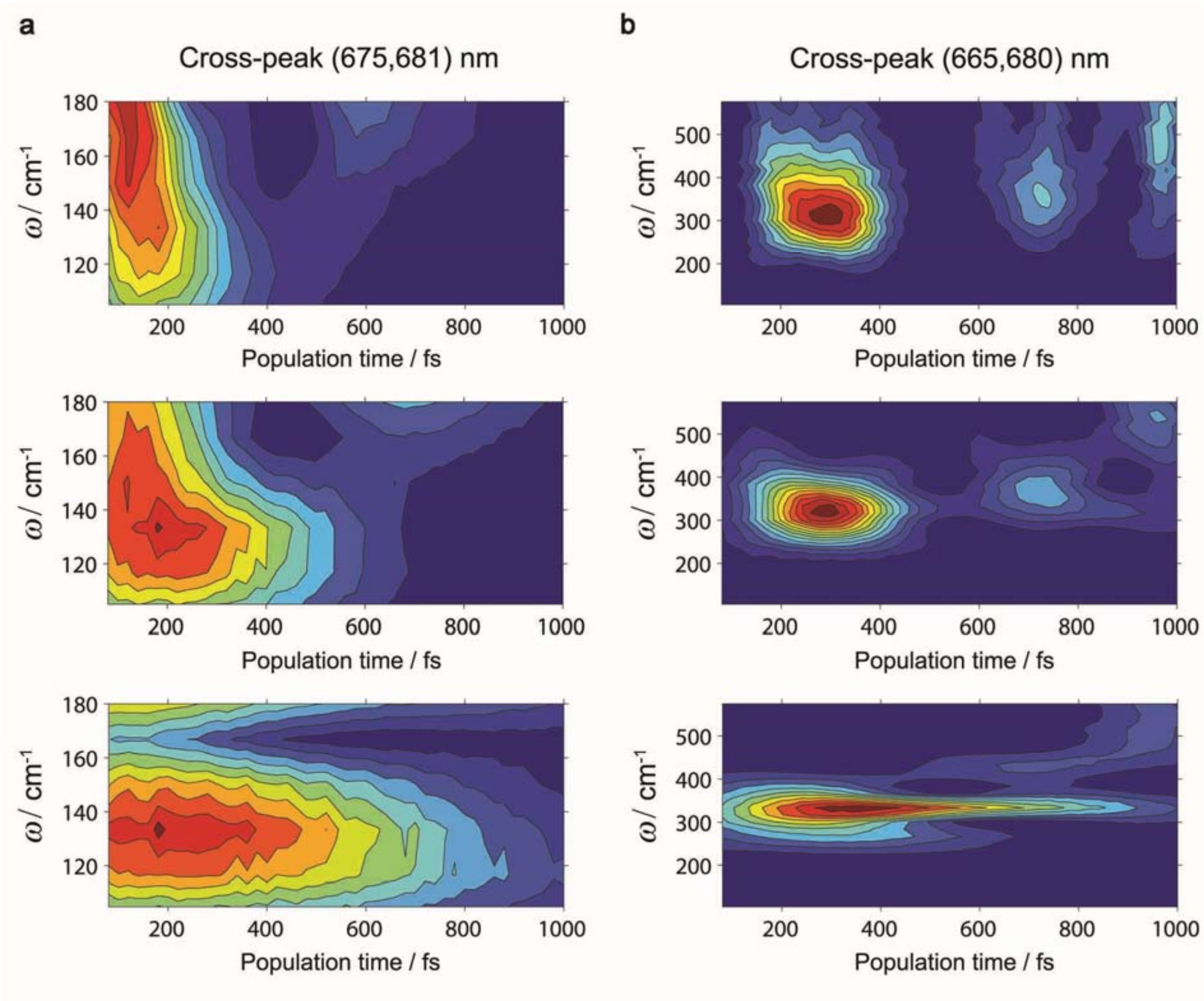

Figure S3. Effect of the F_b value in the frequency/time wavelet resolution relation. (**a**) Scalogram for the main cross-peak in the 120 cm^{-1} 2D frequency map at (675,681) nm: (*top*) F_b = 10, high time resolution, (*middle*) F_b = 2, optimal compromise between time and frequency resolution, (*bottom*) F_b = 0.5, high frequency resolution. (**b**) Scalogram for the (665,680) nm cross-peak in the 340 cm^{-1} 2D frequency map: (*top*) F_b = 10, high time resolution, (*middle*) F_b = 2, optimal compromise between time and frequency resolution, (*bottom*) F_b = 0.5, high frequency resolution.

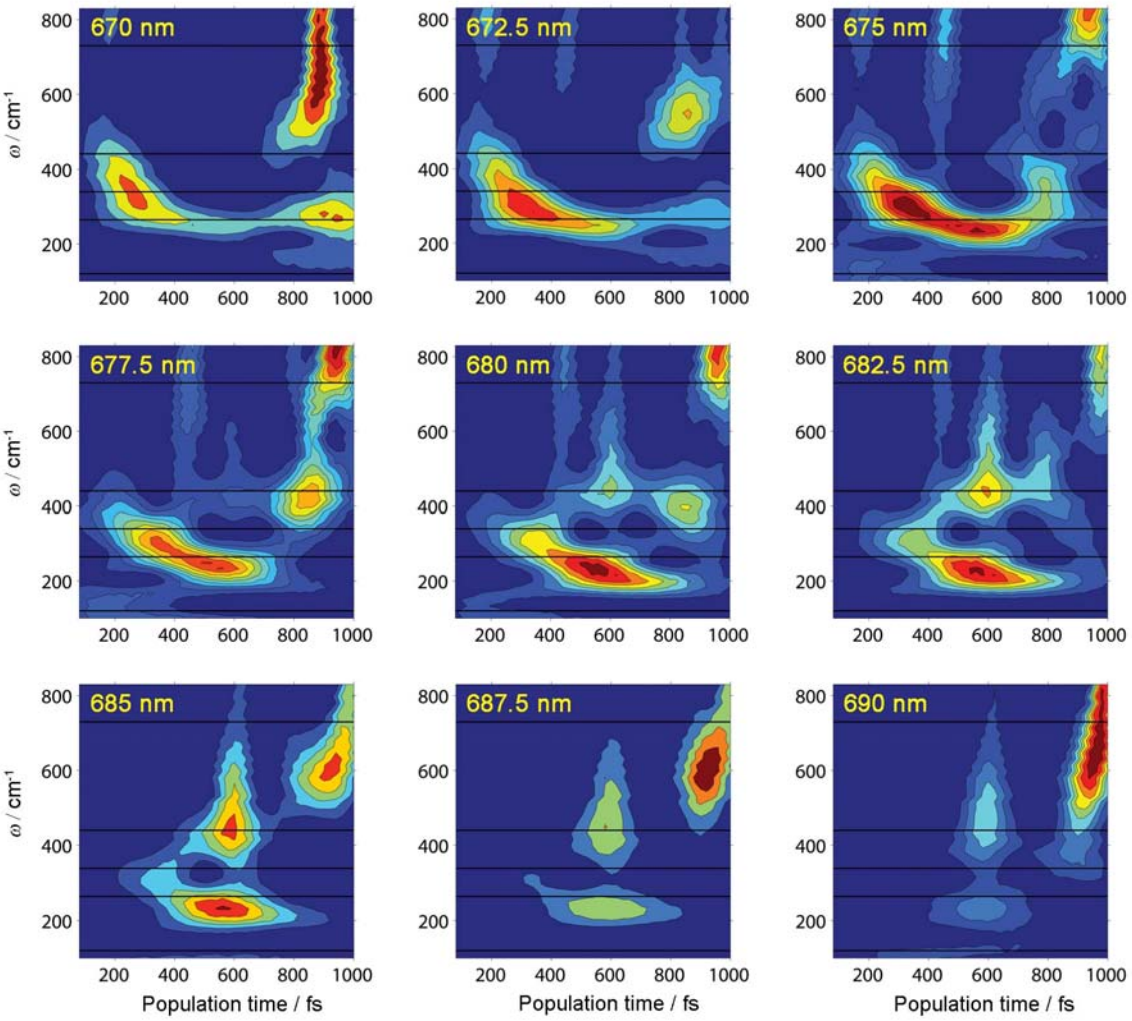

Figure S4. Dynamics in PSII RC at 80 K for the diagonal bands scanned at 2.5 nm intervals. The black horizontal lines indicate the 120, 265, 340 and 440 cm^{-1} oscillation frequencies.

The diagonal bands wavelet data (shown as frequency-time scalograms) shows a complex pattern where the diagonal bands oscillate at several frequencies during different time ranges. This complexity arises from the contribution of vibrational coherences associated with several electronic states (with time-dependent populations), electrochromic shifts induced by charge-transfer and charge-separated states, strong spectral overlap, and presence of a non-trivial interplay between mixed exciton-vibrational coherences[1]. Therefore, with the available 2DES experimental data and the analysis presented here, we conclude that the interpretation of the diagonal bands wavelet data is not possible at this stage and it is out of the scope of the present work. Further experiments and modeling are needed to interpret the diagonal bands wavelet data.

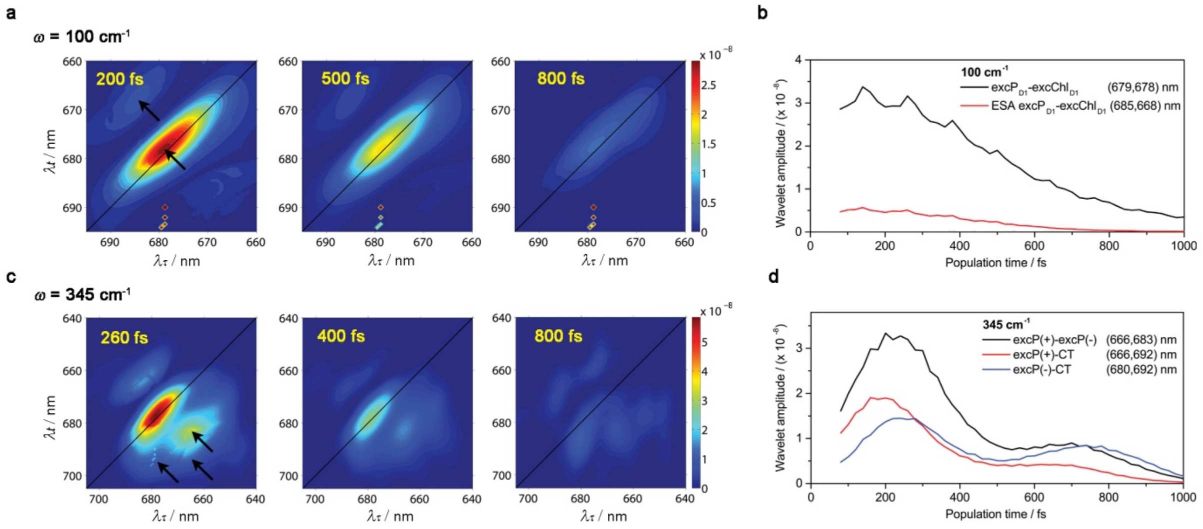

Figure S5. Dynamics of vibronic coherence in the PSII RC at 277K: *time-resolved* 2D frequency maps (a,c) and wavelet traces (b,d). (**a**) 100 cm^{-1} *time-resolved* 2D frequency maps at T = 200, 500 and 800 fs. The black arrows in the 200 fs time-resolved 2D frequency map indicate the position of the selected 2D traces. (**b**) 100 cm^{-1} 2D wavelet traces. (**c**) 345 cm^{-1} *time-resolved* 2D frequency maps at T = 260, 400 and 800 fs. The black arrows in the 260 fs time-resolved 2D frequency map indicate the position of the selected 2D wavelet traces. (**d**) 340 cm^{-1} 2D wavelet traces. The black arrows in the 200 fs time-resolved 2D frequency map indicate the position of the selected 2D wavelet traces.

The dynamics at 277 K are very similar to the dynamics at 80 K presented in Fig. 2 in the main text.

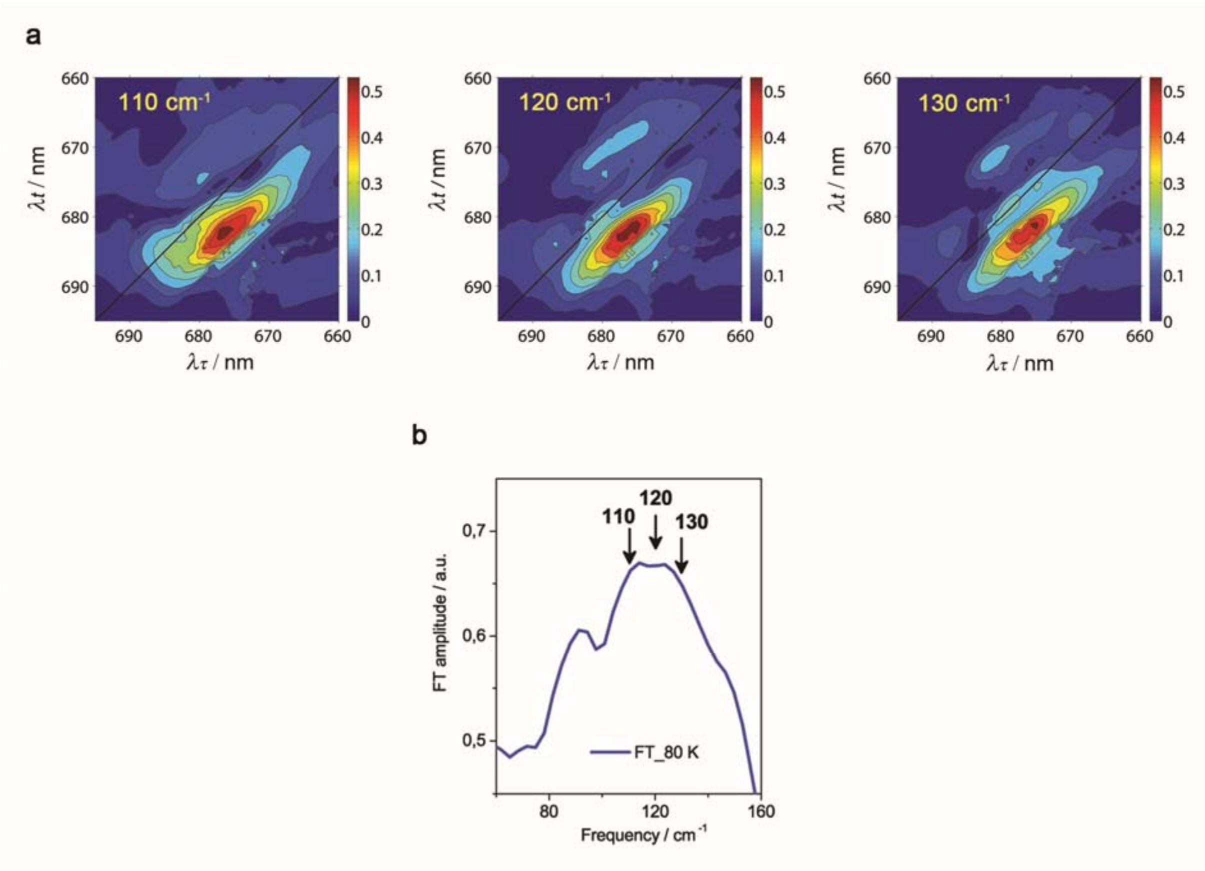

Figure S6. 120 cm^{-1} FT band analysis and 2D frequency maps at 80 K. (**a**) 110, 120 and 130 cm^{-1} 2D frequency maps along the FT band at 80 K. (**b**) FT amplitude of the 120 cm^{-1} band at 80 K. The frequencies displayed in panels a are indicated by arrows. This figure has been adapted from ref. 12 in the main text.

The 80 K FT band centred at 120 cm^{-1} contains two separated maxima at 110 and 130 cm^{-1}. The 110 cm^{-1} 2D frequency map shows one maximum at (677, 683) nm, the 130 cm^{-1} map shows one maximum at (675, 681) nm, whereas the 120 cm^{-1} map sows both maxima at (675, 681) nm and (677, 683) nm. This indicates that the 120 cm^{-1} map represents a superposition of the 110 and 130 cm^{-1} frequencies. Each of these two maxima correspond to coherence between two different realizations of the disorder, the (675, 681) nm maximum corresponds to coherence between $(P_{D2}^{\delta+}P_{D1}^{\delta-})^*_{\approx 675nm}$ and $(Chl_{D1}^{\delta+}Phe_{D1}^{\delta-})^*_{\approx 681nm}$ whereas the (677, 683) nm maximum corresponds to coherence between $(P_{D2}^{\delta+}P_{D1}^{\delta-})^*_{\approx 677nm}$ and $(Chl_{D1}^{\delta+}Phe_{D1}^{\delta-})^*_{\approx 683nm}$ (where the subscripts indicate the central absorption wavelength of each realization of the disorder and the superscripts δ+/δ- indicate CT character). Furthermore, taking into account the width of the diagonal band red shifted 120 cm^{-1} below the diagonal, it should be noted that other realizations where the $(P_{D2}^{\delta+}P_{D1}^{\delta-})^*$ and $(Chl_{D1}^{\delta+}Phe_{D1}^{\delta-})^*$ states absorb at higher and lower energies are also present within that bandwidth.

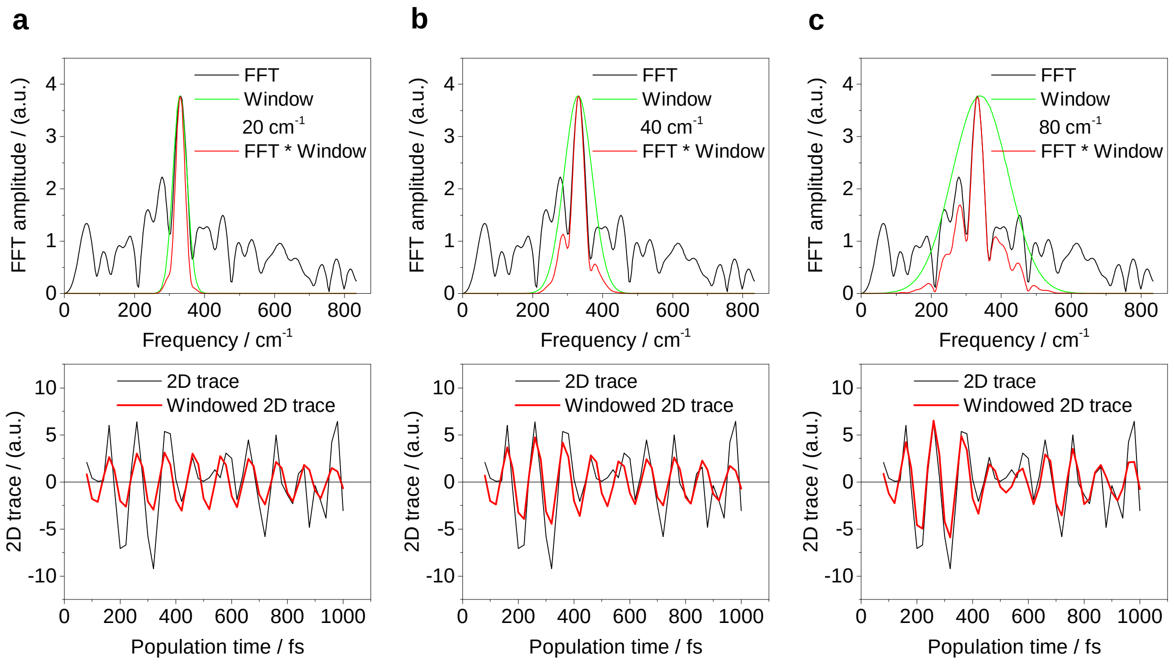

Figure S7. Windowed Fourier transform: comparison of windows with increasing full width at half maximum (fwhm). (*Top*) Fast Fourier transform (FFT) of the (665,680) nm 2D trace at 80 K, window applied with fwhm of 20 (**a**), 40 (**b**) and 80 cm^{-1} (**c**), and the convolution of the FFT with the window. (*Bottom*) (665,680) nm 2D trace (after two exponential decay has been subtracted from the data) and the back FFT of the convoluted windowed FFT (windowed 2D trace).

As explained in the main text, the interference artifact is observed only when closely spaced frequencies are included in the windowed FFT: with a 20 cm^{-1} fwhm window there is no interference present and the 2D trace just shows a decay whereas with a 40 and 80 cm^{-1} fwhm window the interference artifact is clearly present.

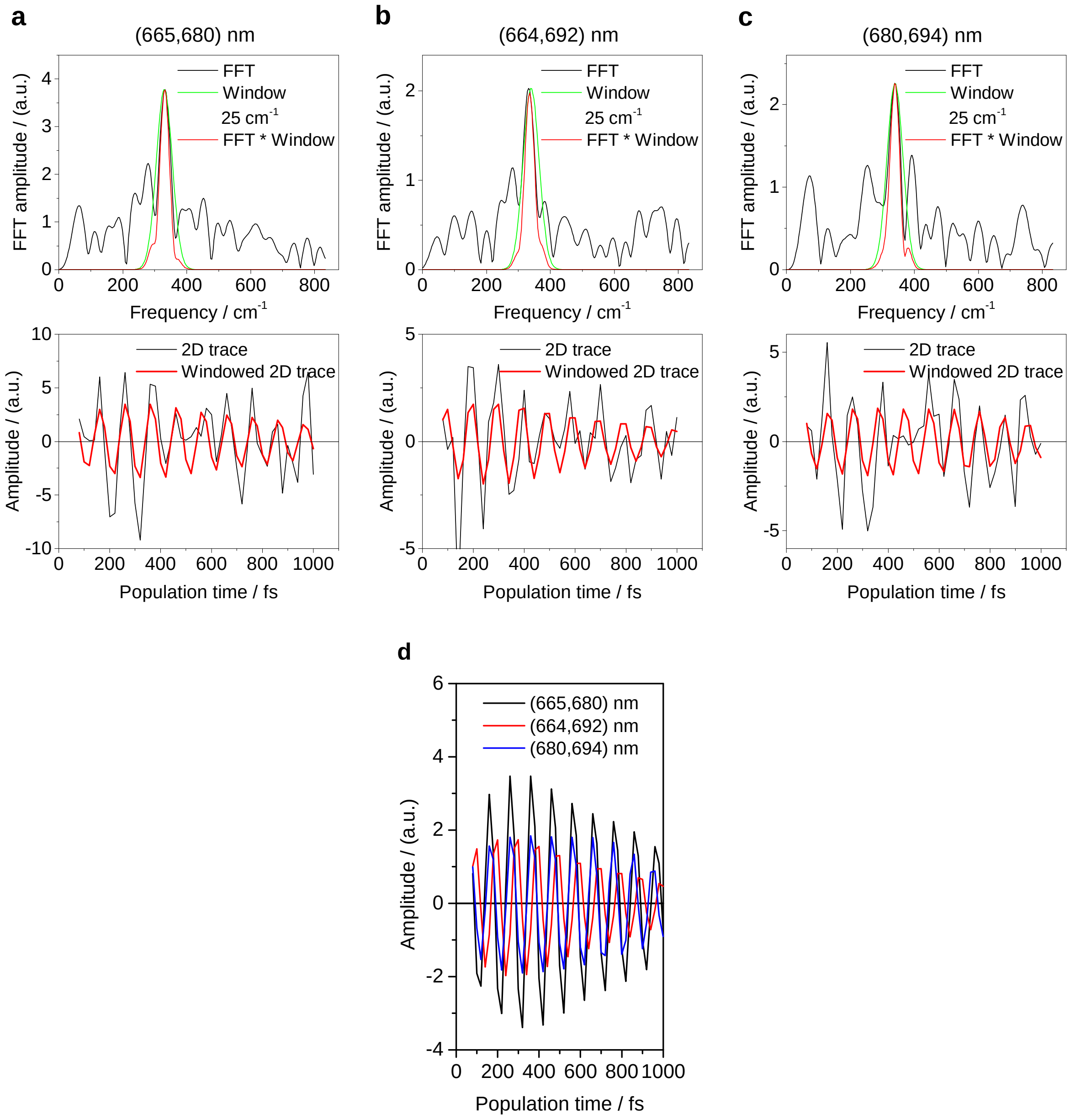

Figure S8. Windowed Fourier transform: cross-peaks below diagonal in the 340 cm^{-1} 2D frequency map with a 25 cm^{-1} (fwhm) window. (*Top*) Fast Fourier transform (FFT) of the (665,680) (**a**), (664,692) (**b)** and (680,694) nm (**c**) 2D traces, window applied with 25 cm^{-1} (fwhm) , and the convolution of the FFT with the window. (*Middle*) (665,680) (**a**), (664,692) (**b**) and (680,694) nm (**c**) 2D traces (after two exponential decay has been subtracted from the data) and the back FFT of the convoluted windowed FFT. (**d**) Comparison of the windowed (665,680), (664,692) and (680,694) nm 2D traces.

Investigating the origins of the amplitude modulations observed in the wavelet data

Here we explore the possible origins of the amplitude modulations observed in the wavelet data further. Specifically, we compare time-frequency signals caused by interferences between different frequencies (interference artefact) with signals which rise/decay due to the creation, regeneration and/or transfer of electronic coherences (*real* dynamics). We show that in principle these signals cannot be distinguished. However the context in which they appear in the 2D spectra, including their position, frequency and a comparison with the known vibrational modes and electronic states in the PSII RC complex can provide further insight on how to distinguish between an interference artefact and *real* dynamics of coherence.

Comparison of artificial signals

Recent work has suggested that vibronic coherences might be regenerated in photosynthetic light harvesting complexes[3]. These coherences can be observed as oscillations in the 2D traces and the dynamics of such coherences are expected to modulate the amplitudes of these oscillations (*real* dynamics). However, since multiple vibrational modes exist in photosynthetic complexes, these modes can interfere with each other, which also leads to a modulation of the oscillation amplitudes (interference artefact).

In order to investigate the differences between these two contrasting signal origins, we consider two artificial signals, given by:

$$A_1(T)=\cos\big((\omega_c+\omega_m)T\big)+\cos\big((\omega_c-\omega_m)T\big) \qquad \text{Eq. 1}$$

$$A_2(T)=[A+\cos(\omega_m T)]\cos(\omega_c T) \qquad \text{Eq. 2}$$

where T is the population time, and ω_c and ω_m are the oscillation frequencies with $\omega_c > \omega_m$.

Eq. 1 gives the superposition of two cosine oscillations at different frequencies, $\omega_c \pm \omega_m$ and represents the case of an artefact, where two oscillation frequencies interfere. On the other hand, Eq. 2 corresponds to the case of *real* dynamics of a coherence: the cosine wave at frequency ω_c represents the vibronic coherent oscillation frequency, whose amplitude is modulated by a cosine wave with frequency ω_m to simulate the effect of regeneration or transfer of the vibronic coherence.

If A = 0, these signals are identical (up to a factor of two in the overall amplitude). They cannot therefore be distinguished from each other in a wavelet analysis. We briefly note that adding arbitrary phases to the two sinusoids in Eq.1 changes the temporal profile of the signal but adding a suitable phase to the envelope and/or carrier factors in Eq.2 can also exactly reproduce this signal (when A=0). Given this inherent ambiguity we ignore such phases in the following. This underlines the heart of the problem: the fact that the oscillation frequency amplitude modulation caused by regeneration of coherences over the population time, *T,* (Eq. 2) is in principle indistinguishable from the amplitude modulation caused by interference between two closely spaced frequencies (Eq. 1). In Figure S9 we show an experimental 2D spectral trace as well as the calculated time signal (analogous to the 2D spectral trace). We also plot the corresponding wavelet pattern (equivalent to the scalograms, the frequency-population time plots) for either $A_1(T)$ or $A_2(T)$ (with A = 0), where $\omega_c = 340\ cm^{-1}$ and $\omega_m = 35\ cm^{-1}$, as an example of the type of interference pattern produced. To perform the wavelet analysis of the calculated time signals we use a Gabor wavelet of frequency 8, with 10 octaves and 15 voices per octave. The data is padded to reduce boundary effects and the sample rate is 20 fs (in line with the experimental data presented in the main text).

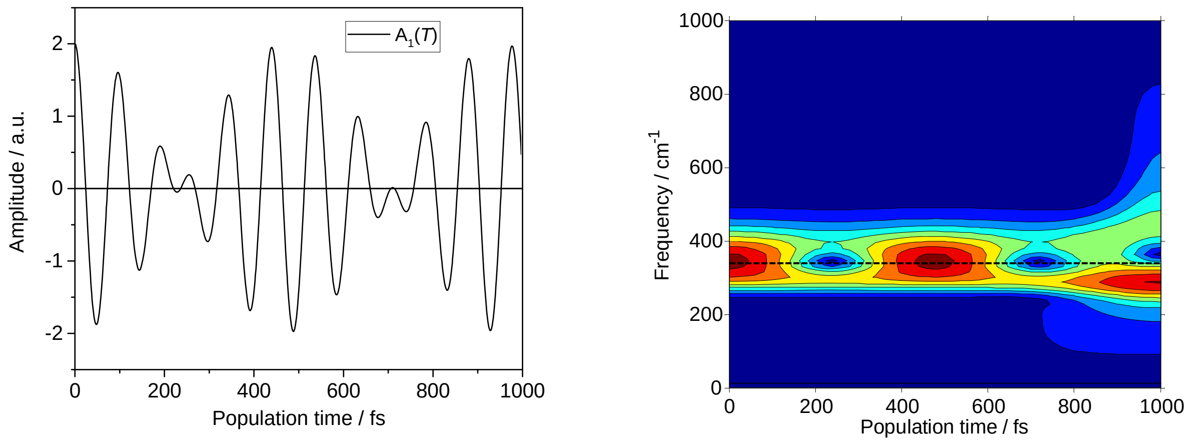

Figure S9. Signal amplitude and corresponding wavelet scalogram for the signal A_1(T), in which two oscillations with different frequencies interfere. (*Left*) $A_1(T)$ plotted for $\omega_c = 340\ cm^{-1}$ and $\omega_m = 35\ cm^{-1}$. (*Right*) Corresponding wavelet signal. Note that these plots are identical to those for $A_2(T)$ with A = 0. The horizontal dashed black line indicates the 340 cm^{-1} oscillation frequency.

Note that for this case, where A = 0, we could also have written a more general, complex form of the equations above as follows:

$$A_3(T) = \exp(i(\omega_c + \omega_m)T) + \exp\big(i(\omega_c - \omega_m)T\big) \qquad \text{Eq. 3}$$

$$A_4(T) = \exp(i\omega_c T)\cos(\omega_m T) \qquad \text{Eq. 4}$$

Here both the real and imaginary parts of the signals are again identical (up to an arbitrary amplitude) and therefore cannot be distinguished. However, in practice it may be possible to distinguish between these types of signals by the context in which they occur, as will be explored below.

1. Population time at which the oscillation frequency amplitude modulation occurs

For Eq. 1, the period at which the *apparent* amplitude modulation occurs due to interference effects is set by the difference between the two frequencies $\omega_c \pm \omega_m$, which is determined by ω_m in our case. In practice, the frequencies at which the 2D spectral amplitude oscillates are known (they are obtained by Fourier transform of the 2D data over the population time), therefore, it is straightforward to calculate such a period (as indicated in the main text). The fact that the observed period of amplitude modulation corresponds to the difference between the two oscillation frequencies is therefore a strong indication that such modulation does not reflect *real* coherent dynamics but an artefact due to interference effects.

For Eq. 2, the oscillation amplitude over the population time depends on both ω_m and A. Note that in the 2D frequency maps the term A in Eq. 2 could be non-zero due to an additional long-lived vibrational coherence which may appear at the same position in the 2D map as the feature corresponding to vibronic coherence. Due to the complexity of multichromophoric systems, the vibronic and vibrational features may overlap in some positions in the 2D frequency maps[1,4-5]. However, we note that this overlap could be avoided if a specific polarisation sequence is applied in the 2DES experiment[6]. Figure S13 shows the effect of changing the amplitude, A, on the time signals and the wavelet analysis, respectively. The results show that changing A defines whether the amplitude of oscillations falls to zero, the population times at which this occurs and the overall amplitude of the amplitude modulations. In this case, it is not straightforward to distinguish between *real* and *apparent* coherence dynamics.

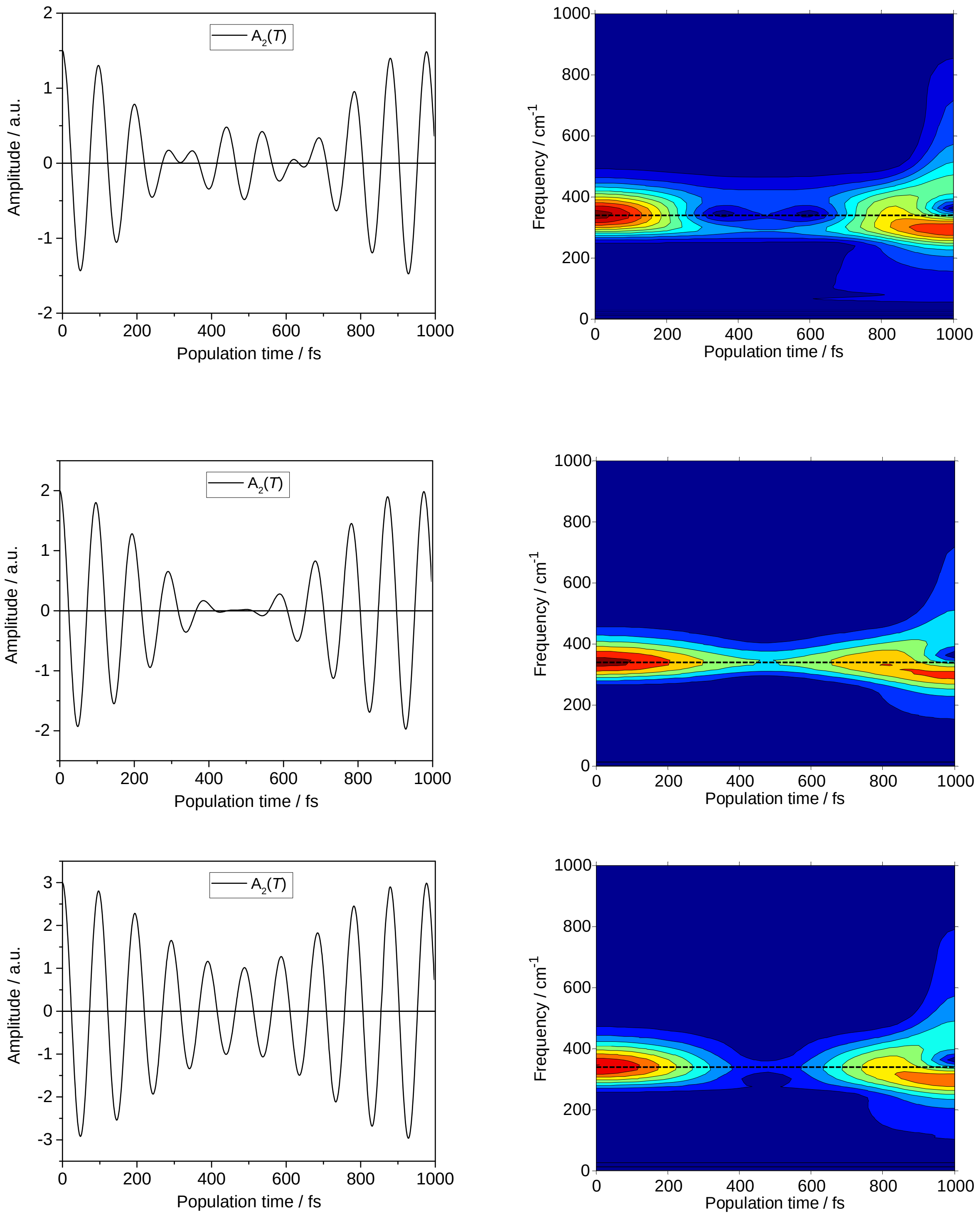

Figure S13. Signal amplitudes and corresponding wavelet scalograms for the signal A_2(T) with different values of the constant A. (*Left panels*) $A_2(T)$ plotted for ω_c = 340 cm^{-1} and ω_m = 35 cm^{-1} with (*top*) A = 0.5, (*middle*) A = 1, (*bottom*) A = 2. (*Right panels*) Corresponding wavelet signal with (*top*) A = 0.5, (*middle*) A = 1, (*bottom*) A = 2. The horizontal dashed black line indicates the 340 cm^{-1} oscillation frequency.

Changing position in 2D spectrum

Here we consider how the 2D wavelet traces vary as a function of the position (ω_τ, ω_t) in the 2D frequency maps. In the 2D spectra, the diagonal peaks are expected to contain a superposition of states which oscillate at different frequencies and thus create a complicated pattern of interfering frequencies (interference artefact) overlapped with potential *real* coherence dynamics. In this case, advanced modelling of the experimental data is required to disentangle the different contributions in the observed signal. However, the areas above and below the diagonal are less prone to spectral congestion. For instance, the vibronic coherence between states A and B (absorbing at ω_A and ω_B, respectively, with $\omega_A > \omega_B$) appears in the (ω_A - ω_B) 2D frequency map with a maximum amplitude at position (ω_A, ω_B), and the weight of this frequency decreases when moving away from position (ω_A, ω_B). Using this rationale, here we investigate the effect of the varying contributions of different frequencies in the wavelet pattern (or wavelet traces) when moving away from a cross-peak maximum. For simplicity, we take a fixed ω_τ and a varying ω_t.

Firstly, we consider signals arising from interference between two frequencies, as in Eq. 1, which can be generalised to:

$$A_1^{'}(T)=\alpha\cos\left((\omega_c+\omega_m)T\right)+\beta\cos\left((\omega_c-\omega_m)T\right) \qquad \text{Eq. 5}$$

where we introduce α and β, which allow us to vary the weight of contributions from different oscillation frequencies. As the weighting of the two cosine waves changes, the amplitude of the pattern observed in the wavelets will shift to different frequencies along the y-axis, as shown in Figure S11. However the population time at which the recurrence occurs remains the same (as determined by ω_m; see above). Also notice that interference between waves at two different frequencies can cause oscillations in the amplitude of the wavelet even if one of the amplitudes α or β is significantly larger than the other and it is not obvious from the wavelet results that two different frequencies are involved.

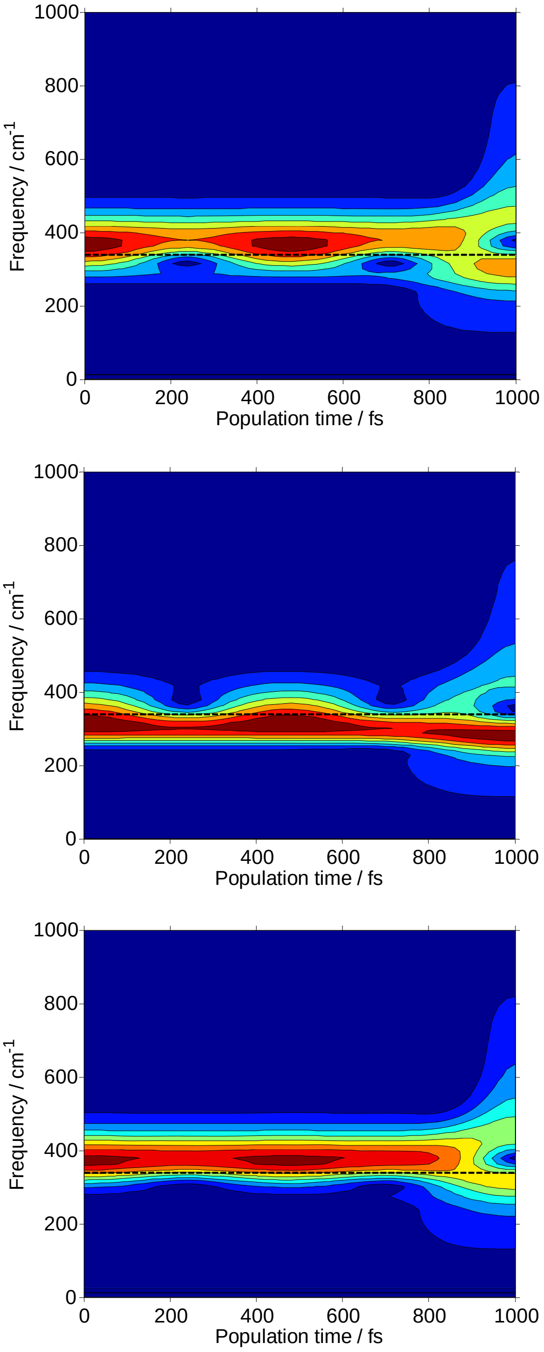

Figure S11. Wavelet scalograms for the signal A₁'(T) with different weightings of *α* and *β*. Wavelet signal for $A_1'(T)$ with $\omega_c = 340$ cm^{-1} and $\omega_m = 35$ cm^{-1} and (*top*) $\alpha = 0.75$ and $\beta = 0.25$, (*middle*) $\alpha = 0.25$ and $\beta = 0.75$ and (*bottom*) $\alpha = 0.9$ and $\beta = 0.1$. The horizontal dashed black line indicates the 340 cm^{-1} oscillation frequency.

For amplitude modulation of the type given by Eq. 2, changing ω_c will change the frequency (on the y axis) at which the pattern in the wavelets occurs but not the weighting of the pattern itself; see Figure S12. Therefore shifts in the weighting of the pattern may indicate that the signal arises from interference between different frequencies (as described above).

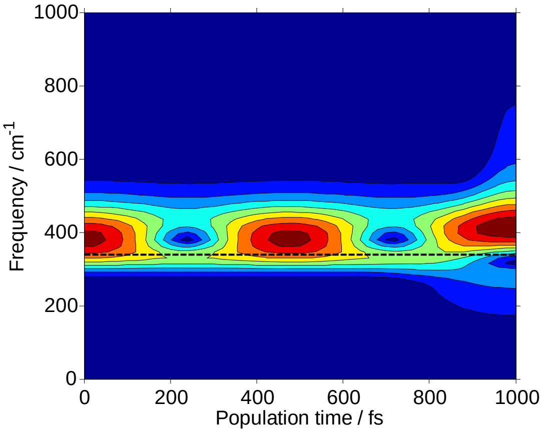

Figure S12. Wavelet scalogram for the signal $A_2(T)$. Wavelet signal for Eq. 2 with ω_c = 380 cm^{-1} and ω_m = 35 cm^{-1} and A = 0. The frequency on the y-axis shifts compared to Fig. 1, but the pattern over the population time is unchanged. The horizontal dashed black line indicates the 340 cm^{-1} oscillation frequency.

Conclusions

In conclusion, by considering artificial signals we have shown that the time signal from two interfering oscillations with different frequencies can be identical to the signal from amplitude modulation of an oscillation which might arise from regeneration of a vibronic coherence. Therefore in principle these two different signal origins cannot be distinguished, either by a wavelet analysis or any other technique.

However, 2D spectroscopy provides a wealth of information beyond that which is contained in a single time, therefore this additional information can give further insight into the signal dynamics origin. In particular, in the case of interference between two oscillations with different frequencies, the period of the interfering envelope is determined by the frequency difference between the oscillations. This means that the period of the oscillations expected from this effect can be calculated and compared to the experimental results (as in the main text). We note that care should be taken if there are overlapping signals in the 2D spectra (for example, from ground-state bleach), since these can change the amplitude and period of the oscillations observed. In that case, it may be helpful to simulate the signals in order to make quantitative predictions.

In addition, we highlight that interference between waves at two different frequencies can cause oscillations in the amplitude of the wavelet even if one of the oscillations has a significantly larger amplitude than the other. In this case it may be very difficult to discern from a visual inspection of the wavelet results that two different frequencies are interfering.

Finally, changing the position considered in the 2D spectrum is expected to have different effects depending on the signal origin. Notably, as the position in the 2D spectrum changes, for interference effects the weighting of the pattern observed in the wavelet scalograms is expected to shift, whereas for the amplitude modulation expected from electronic coherences, the period of the modulation changes.

References

1 Novoderezhkin, V. I., Romero, E. & van Grondelle, R. How exciton-vibrational coherences control charge separation in the photosystem II reaction center. *Phys. Chem. Chem. Phys.* **17**, 30828-30841 (2015).

2 Novoderezhkin, V., Romero, E., Prior, J. & van Grondelle, R. Exciton-vibrational resonance and dynamics of charge separation in the photosystem II reaction center. *Phys. Chem. Chem. Phys.*, doi:10.1039/c6cp07308e (2017).

3 Chin, A. W. *et al.* The role of non-equilibrium vibrational structures in electronic coherence and recoherence in pigment-protein complexes. *Nat. Phys.* **9**, 113-118 (2013).

4 Butkus, V., Zigmantas, D., Valkunas, L. & Abramavicius, D. Vibrational vs. electronic coherences in 2D spectrum of molecular systems. *Chem. Phys. Lett.* **545**, 40-43 (2012).

5 Ferretti, M. *et al.* The nature of coherences in the B820 bacteriochlorophyll dimer revealed by two-dimensional electronic spectroscopy. *Phys. Chem. Chem. Phys.* **16**, 9930-9939, doi:10.1039/c3cp54634a (2014).

6 Schlau-Cohen, G. S. *et al.* Elucidation of the timescales and origins of quantum electronic coherence in LHCII. *Nat. Chem.* **4**, 389-395 (2012).
